# Supplementary material for: Missense mutations on SynGAP C2 domain impair membrane diffusion
Source: Protein Sci. 2026 Apr 15;35(5):e70574. doi: 10.1002/pro.70574 (PMC13081684; doi:10.1002/pro.70574)
Supplement: Supplementary file 1 — Figure S1. Bar plot representation of the PCA loadings. Figure S2. Scatter plot of covariance matrix elements for the motion of the C2 domain when isolated vs. when it is embedded in the SynGap structure. Table SI. List of known benign (B) or likely benign (LB) C2 single mutations. Table SII. List of known pathogenic (B) or likely pathogenic (LB) C2 single mutations. Table SIII. List of known variance under surveillance (VUS) of the C2 domain. Table SIV. List of known C2 single mutations with conflicting effect. [file PRO-35-e70574-s001.pdf]

Supplementary Information - Missense mutations on SynGAP C2 domain impair membrane diffusion

Mattia Miotto*,[1,2] Leonardo Bo',[2] Giancarlo Ruocco,[2,1] Silvia Di Angelantonio,[2,3] and Bernadette Basilico*[2,3]

[1]*Department of Physics, Sapienza University of Rome, Rome, Italy*
[2]*Center for Life Nano & Neuro Science, Italian Institute of Technology, Rome, Italy*
[3]*Department of Physiology and Pharmacology, Sapienza University of Rome, Rome, Italy*

I. ADDITIONAL TABLES AND FIGURES

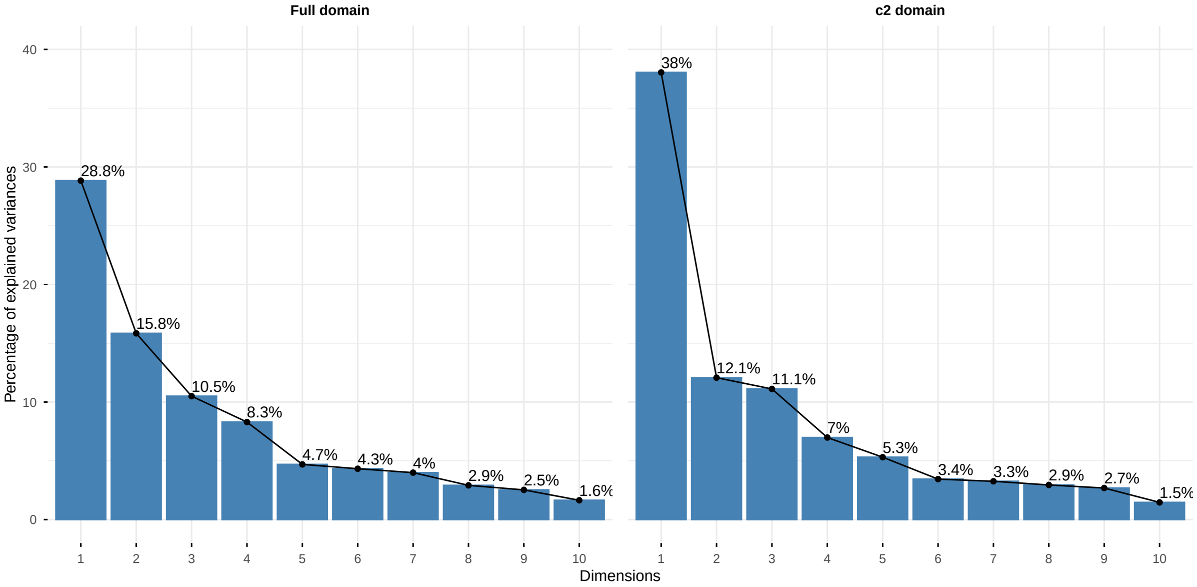

FIG. 1: Bar plot representation of the PCA loadings.

* Corresponding authors:
mattia.miotto@roma1.infn.it; bernadette.basilico@uniroma1.it

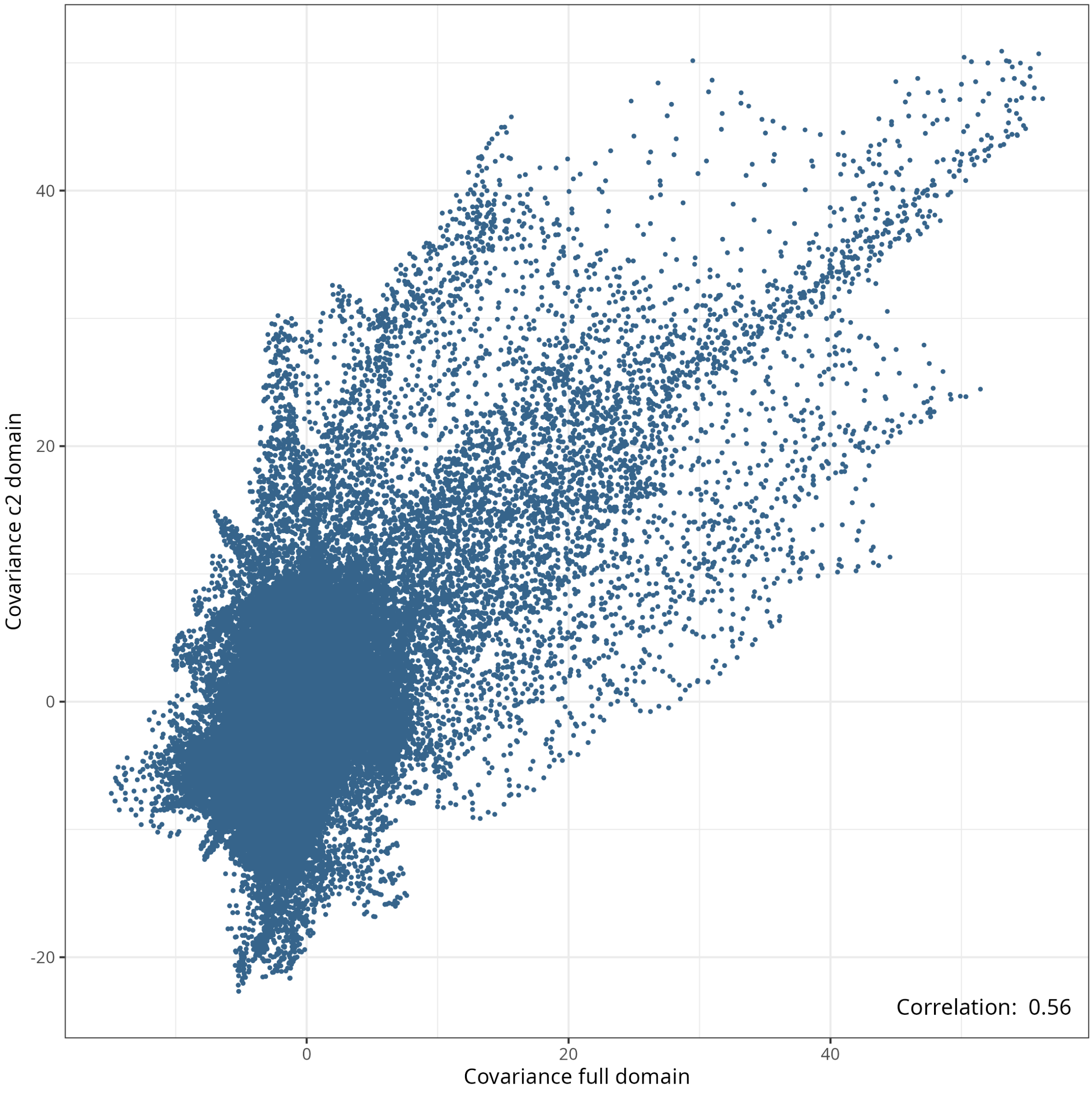

FIG. 2: Scatter plot of covariance matrix elements for the motion of the C2 domain when isolated vs when it is embedded in the SynGap structure.

Benign/Likely benign	Mutation	
1	R258H	B/LB
2	E273D	B
3	M289V	B
4	T352N	LB
5	R356C	LB
6	S379L	B
7	S385W	B
8	A399T	B
9	V400L	B

TABLE I: List of known benign (B) or likely benign (LB) C2 single mutations.

Patogenic/Likely Patogenic	Mutation	
1	A271D	P
2	C282R	P
3	D287H	LP
4	D287Y	LP
5	R293P	LP
6	W308C	P/LP
7	W308R	P
8	E310D	LP
9	L323R	LP
10	L327P	P
11	G344S	P
12	W362R	P
13	L402R	LP

TABLE II: List of known pathogenic (B) or likely pathogenic (LB) C2 single mutations.

VUS	Mutation
1	R259Q
2	R259W
3	L264Q
4	R272Q
5	L274Q
6	R279W
7	C282S
8	Y291C
9	R293C
10	S296A
11	S300F
12	A301T
13	T305A
14	V306D
15	P318L
16	V320L
17	R321H
18	L323P
19	R324W
20	R329H
21	R335H
22	Y342S
23	V343I
24	V348M
25	P349S
26	G370S
27	G373V
28	S374Y
29	S379W
30	G381V
31	G384S
32	S385L
33	S385P
34	G387V
35	G390E
36	G391V
37	P398L
38	V400E

TABLE III: List of known variance under surveillance (VUS) of the C2 domain.

Conflicting	Mutation
1	R299C
2	R299H
3	E310K
4	Y342C
5	R405C
6	R405H

TABLE IV: List of known C2 single mutations with conflicting effect.
